# Buffalo Academy of Medicine

**Published:** 1906-04

**Authors:** Franklin W. Barrows


### SOCIETY PROCEEDINGS.

### Buffalo Academy of Medicine

Reported By FRANKLIN W. BARROWS, M. D.

*Section of Medicine, February 20, 1906.*

THE regular meeting of the Section of Medicine was held in the Academy Rooms, Public Library Building, on Tuesday, February 20, 1906, the chairman Dr. A. W. Bayliss, presiding. The section was called to order at 9.00 p. m. The minutes of the previous meeting were read and approved.

The first paper, entitled "Longevity among Yale Athletes," was presented by Dr. William Gilbert Anderson, M.A., Director of the Yale University Gymnasium. The paper included many interesting statistics of Yale athletes from 1855 to 1905. His figures led him to the conclusion that, in general, Yale athletes do not die of heart disease, that they live to a good age, and that their athletic work seems to have a favorable effect on longevity.

Dr. Herbert U. Williams opened the discussion and spoke particularly of the effect of athletic stress and strain on the arterial system. Dr. J. W. Grosvenor inquired as to the influence of heredity and abstinence on the longevity of athletes. Dr. Irving Lyon thought it would be very profitable to make careful observations of blood-pressure in athletes at the beginning and the end of their career. Dr. Anderson said, in conclusion, that careful observations of blood-pressure had been practised in the Springfield, Mass., Y. M. C. A. and the Yale Gymnasium, from which we may look for valuable results. He spoke also of the strict abstinence and regular habits required of men in training for athletic honors.

The second paper, entitled "Functional Derangement of the Heart," was presented by Dr. James S. Smith, who discussed a number of cases that have occurred in his practice.

At 10.20 the meeting adjourned to March 6. Attendance, 41.

*Section of Medicine, March 6, 1906.*

Reported By HENRY E. STADLINGER, M. D., Secretary Pro tem.

The meeting was called to order at 9 p. m. March 6, 1906, the chairman, Dr. A. W. Bayliss, presiding. In the absence of Dr. Barrows, secretary, Dr. Henry E. Stadlinger acted as secretary, *pro tem.*

The minutes of the previous meeting were read and approved. The first paper, on "Therapeutic Treatment of Pneumonia" was read by Dr. George H. Westinghouse, in which he outlined the general treatment of pneumonia. The second paper was read by Dr. G. Tartaro, and was entitled "Report of 25 Cases of Lobar Pneumonia treated with Antipneumonic Serum." The doctor emphasized the importance of using Dr. Pane's serum, imported from Italy, and spoke of the absolutely useless antipneumonic serums made in this country.

The papers were discussed together. Dr. Grosvenor opened the discussion and made a plea against the use of alcohol in pneumonia. Dr. Jewett said good results were obtained from bloodletting, and particularly in alcoholic cases. Drs. Hartwig, Thoma, Blaauw, and others also entered into the discussion.

Adjourned at 10.30. Attendance 19.

At the close of the meeting Dr. Howe brought before the Academy the "Local Option Bill" pending at Albany, and his motion concerning it was unanimously carried, Dr. Howe being named to notify the local representative at Albany.

---

